# DNA Methylation, Nuclear Organization, and Cancer

**DOI:** 10.3389/fgene.2017.00076

**Published:** 2017-06-07

**Authors:** Bhavani P. Madakashira, Kirsten C. Sadler

**Affiliations:** Program in Biology, New York University Abu Dhabi,Abu Dhabi, United Arab Emirates

**Keywords:** chromatin, DNA methylation, lads, Large Organized Chromatin Lysine Modifications (LOCKS), cancer

## Abstract

The dramatic re-organization of the cancer cell nucleus creates telltale morphological features critical for pathological staging of tumors. In addition, the changes to the mutational and epigenetic landscape in cancer cells alter the structure and stability of the genome and directly contribute to malignancy. DNA methylation is one of the best studied epigenetic changes in cancer, as nearly every type of cancer studied shows a loss of DNA methylation spread across most of the genome. This global hypomethylation is accompanied by hypermethylation at distinct loci, and much of the work on DNA methylation in cancer has focused on how local changes contribute to gene expression. However, the emerging picture is that the changes to DNA methylation in cancer cells has little direct effect on gene expression but instead impacts the organization of the genome in the nucleus. Several recent studies that take a broad view of the cancer epigenome find that the most profound changes to the cancer methylome are spread across large segments of the genome, and that the focal changes are reflective of a whole reorganization of epigenome. Hallmarks of nuclear reorganization in cancer are found in the long regions of chromatin marked by histone methylation (LOCKs) and nuclear lamina interactions (LADs). In this review, we focus on a novel perspective that DNA methylation changes in cancer impact the global structure of heterochromatin, LADs and LOCKs, and how these global changes, in turn, contribute to gene expression changes and genomic stability.

## Introduction

For over a century, pathologists have used cellular morphology to guide their diagnosis of cancer. Chief among the morphological features that distinguish benign from malignant cells is the shape, size, structure and composition of the nucleus (Zink et al., 2004). Moreover, nuclear morphologies are often pleiotropic across a single tumor, reflecting the heterogeneous nature of cancer. Altered nuclear morphology also reflects broad changes in genome positioning and epigenetic changes which occur during transformation. We review recent data showing that widespread epigenetic changes are among the most prominent and common features of the cancer nucleus.

Chromatin organization is dictated by interactions between DNA and nuclear structural proteins, such as nuclear lamins, and by epigenetic modifications. These modifications and changes to nuclear structure are key features distinguishing cancer cells from their normal counterparts. Moreover, changes to genome structure and epigenetic changes also can be tumorigenic, by causing genomic instability, a hallmark of cancer (Hanahan and Weinberg, 2011).

Here, we discuss data showing that three epigenetic marks that occupy large regions of the genome are changed in cancer. First, cytosine methylation (5mC) of DNA is among the most well studied epigenetic modifications, global loss of DNA methylation is a common feature of cancer (Feinberg and Vogelstein, 1983a; Gama-Sosa et al., 1983). Recent work has shown that the pattern of DNA hypomethylation in cancer is characterized by long regions termed Partially Methylated Domains (PMDs) (Lister et al., 2009; Hon et al., 2012), accompanied by massive disruption in nuclear organization. The Lamina Associated Domains (LADs) (Guelen et al., 2008) and the long stretches of the genome termed Large Organized Chromatin lysine (“K”) modification (LOCKs) (Wen et al., 2009; McDonald et al., 2011) differ markedly between cancer and normal cells (McDonald et al., 2011; Timp et al., 2014). Exciting work has uncovered significant overlap between PMDs, LADs and LOCKs (Timp and Feinberg, 2013; Timp et al., 2014), suggesting that regional, not local, changes are the defining features of the cancer epigenomic landscape.

## DNA Methylation: Features, Functions and Changes in Cancer

DNA methylation was the first epigenetic change to be reported in cancer (Feinberg and Vogelstein, 1983a; Gama-Sosa et al., 1983; Ehrlich et al., 1985). Over 80% of CpGs are methylated in human somatic cells, these are concentrated in repetitive sequences in intergenic regions and introns, whereas the CpG islands (CGI) found in the promoters of most genes (Lister et al., 2009; Roadmap Epigenomics et al., 2015) are protected from methylation (Deaton and Bird, 2011). This pattern is largely constant across somatic cell types from the same organism, with less than 20% of all CpGs showing any changes in methylation across cell types (Lister et al., 2009; Ziller et al., 2013; Zhang et al., 2016).

**FIGURE 1:**
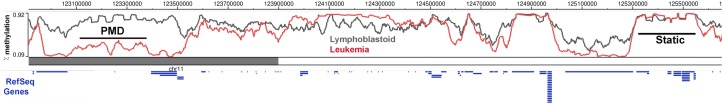
DNA methylation is dynamically altered in cancer cells compared to the normal cells. DNA methylation in a region of chromosome 11 between Lymphoblastoid cells (GM12878) and Leukemia cells (K562) shows regions of partially methylated domains (PMD) in the cancer cells, where the methylation levels flatten at around 50% over the region compared to the DNA methylation of the same region in the control sample. There are also regions which do not show any methylation differences (Static) and a DNA block with hypermethylation in the cancer cells. Ref Seq Genes in the region are represented as blue lines below the histogram. This figure was generated using the data from the Epigenome browser of the Roadmap Epigenomics Project (http://epigenomegateway.wustl. edu/browser/).

Pioneering work showing the essential role of DNA methylation in silencing imprinted genes (Reik et al., 1987; Sapienza et al., 1987; Swain et al., 1987; Li et al., 1993), inactivating X chromosome (Mohandas et al., 1981) and repressing repetitive DNA to prevent transposon activation (Chandler and Walbot, 1986; Schwartz and Dennis, 1986) coincided with the discovery of DNA hypomethylation in cancer (Feinberg and Vogelstein, 1983a; Gama-Sosa et al., 1983). These converged into a model proposing that DNA methylation regulated expression of oncogenes and tumor suppressors. Thousands of studies have pursued this theory; while some convincingly show a direct and inverse relationship between gene expression and DNA methylation of a regulatory region, most do not. In fact, most methylated CpGs reside in intergenic regions and most of the differentially methylated regions (DMRs) between normal and cancer cells are in these regions (Baylin and Jones, 2011). Moreover, the DMRs in cancer are largely not focal, but are instead spread across broad regions of the genome (**Figure 1**). In some cases, these DMRs represent a partial loss of methylation across a large region (i.e., PMD). Thus, the highly cited examples where methylation of a regulatory region controls gene expression in cancer appear to be the exception rather than the rule. We postulate that methylome reorganization in cancer reflects a massive change in the distribution of heterochromatin and nuclear organization, and that this reorganization can indirectly control gene expression.

### The Cancer Methylome and Nuclear Organization

The methylome landscape in normal cells is that of broad peaks across long stretches of intergenic repeat-rich regions and valleys in CGIs and CpG poor regions (**Figure 1**). A birds-eye view of the methylome reveals that the large hypermethylated peaks, become hypomethylated in several cancer types (Varley et al., 2013; Ziller et al., 2013; Landau et al., 2014). Peaks of methylation in normal cells are characterized by stretches with over 80% methylation of most CpGs. These are converted to PMDs (**Figure 1**; Hansen et al., 2011), defined as a 5 kb to 10 Mb region with average 50% CpG methylation (Lister et al., 2009). The emergence of PMDs essentially flattens the methylation landscape in cancer cells, blurring the boundaries between what in normal cells constitute methylation valleys (CGIs) and peaks (CGI shores). The novel concept here is that cancer DMRs (cDMRs) are largely comprised of PMDs, and that the focal regions of differential methylation need to be viewed in the context of the methylation level of the entire region of the genome. An additional novel idea from these studies is that the dramatic change in the cancer methylome is reflective of a randomization of methylation patterns, whereby each cytosine is methylated in some cells and not methylated in others (Hon et al., 2012; Landau et al., 2014; Timp et al., 2014).

The importance of PMDs in cancer was highlighted by a pioneering study focused on “CpG shores.” These are 2 kb away from CGIs, cover genes expressed in a tissue-specific manner, and are variably methylated during tissue differentiation (Irizarry et al., 2009). While most of the methylome is static, shores are the most variable across cell types (**Figure 1**). Interestingly, cDMRs are also found largely in CpG shores (Hansen et al., 2011). Indeed, the location of cDMRs reflects the cell of cancer origin, possibly because these are the regions of the methylome that are most amenable to change.

### DNA Methylation and Cancer: Cause or Consequence?

Genomic instability is carcinogenic. Since regions of DNA hypomethylation in cancer cells correspond with hotspots of chromosomal breaks (Eden et al., 2003; Rodriguez et al., 2006; Irizarry et al., 2009) and regions of the cancer genome where the methylation pattern appears random are more prone to mutation (Landau et al., 2014) suggests a link between DNA hypomethylation and genomic instability. Moreover, repressing transposons is a central function of DNA methylation, and transposon activation can contribute to genomic rearrangements by retrotransposition (Helman et al., 2014; Tubio et al., 2014). Contrasting data from the analysis of 51 premalignant lesions which showed that large blocks of hypomethylation that are found in advanced cancers are detected even in lesions that are not considered prone to malignant transformation (Timp et al., 2014). Further study is required to determine whether DNA hypomethylation can cause cancer in all cell types.

Data from model organisms supports the hypothesis that loss of DNA methylation is oncogenic. In mice, a strong hypomorphic allele of the DNA methyltransferase, *Dnmt1*, causes DNA hypomethylation and genomic instability leading to aggressive T-cell lymphoma (Gaudet et al., 2003) and heterozygosity for this allele synergized with *Nf1* and *Tp53* to accelerate sarcoma formation (Eden et al., 2003). Similarly, a hypomorphic allele of *Dnmt3b* in combination with defects in a DNA repair gene caused lymphoma (Trinh et al., 2002). Our work in zebrafish (Mudbhary et al., 2014) demonstrated that overexpression of the epigenetic modifier, UHRF1, caused liver cancer in the absence of any other sensitizing mutations. In this model, UHRF1 overexpression caused global DNA hypomethylation and chromatin reorganization which first caused senescence as a tumor suppressive mechanism and when senescence was bypassed, tumors formed. We speculate that senescence induced by UHRF1 overexpression may be related to reorganization of the nuclear lamina, which causes senescence in other systems (Criscione et al., 2016).

## Nuclear Organization and Chromatin Domains: LADs and LOCKs

**FIGURE 2:**
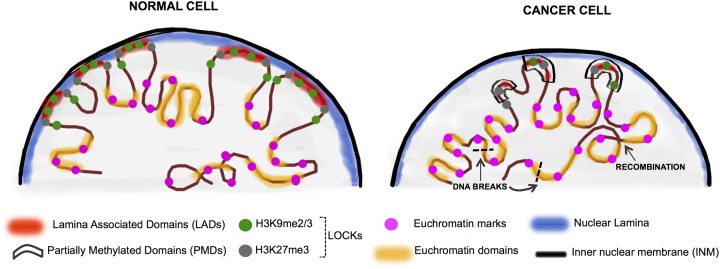
Chromatin rearrangement in the cancer nuclei. In normal differentiated cells, heterochromatin is organized in the nuclear periphery by binding to the nuclear lamina proteins (in blue) and is organized into LADs (red) which overlap significantly with LOCKs (H3K9me2/3 and H3K27me3-green and gray circles). The active domains are tagged by the euchromatin histone marks (pink circles). Cancer cells display nuclear chromatin rearrangement with decreased lamin expression in the lamina, increased euchromatinization, and significant loss of LADs and LOCKs. In cancer, large blocks of DNA termed PMDs (black boxes) coincide to a large extent with LADs and LOCKs. These events can ultimately lead to random DNA recombination events as well as the less stable open chromatin are hotspots for DNA breaks (black dashed lines) (Kind et al., 2013; Luperchio et al., 2014; Feinberg et al., 2016).

Nuclear lamins have essential roles in maintaining nuclear structure, organizing chromosome territories (Cremer et al., 2006), interacting with nuclear actin (Burke and Stewart, 2013) and regulating gene expression (Reddy et al., 2008). A major role for lamins is to interact with distinct regions of the genome (i.e., LADs) covering 35–40% of the mammalian genome in blocks ranging from 0.1 to 10 MB (Guelen et al., 2008; Meuleman et al., 2013; Amendola and van Steensel, 2015). LOCKs were first defined by long stretches of histone H3 lysine 9 dimethylation (H3K9me2) which largely overlap with LADs (Guelen et al., 2008; Hawkins et al., 2010; Peric-Hupkes et al., 2010; Hon et al., 2012; Luperchio et al., 2014; **Figure 2**). Thus, LOCKs represent blocks of the genome which are packaged into repressive chromatin structures. Interestingly, PMDs in cancer largely correspond to regions of LOCKs and LADs (Pujadas and Feinberg, 2012; Luperchio et al., 2014; **Figure 2**).

### LADs and TADs

Most transcription occurs in the center of the nucleus; regions of the genome relegated to the nuclear periphery and encompassed in LADs are generally repressed (Dekker, 2008). Experimental repositioning of genes by targeting them to the nuclear envelope transmembrane proteins leads to repression of key genes involved in myogenesis, demonstrating the importance of gene positioning for tissue specific gene regulation (Robson et al., 2016). Similarly, depletion of B- type lamins in *Drosophila* de-repressed genes at the nuclear periphery (Shevelyov et al., 2009).

Lamina interactions are AT rich, containing epigenetic marks H3K9me2, reduced H3K36me3, with the LAD borders enriched for CTCF, H3K4me3 and H3K27me3 (Guelen et al., 2008; Zullo et al., 2012; Meuleman et al., 2013; Harr et al., 2015). Similar to the finding that most regions of DNA methylation are static across cell types, many LADs also tend to be constitutive across cell types (cLADs), although some are cell type specific. (Guelen et al., 2008; Meuleman et al., 2013). cLADs range from 100 kb to 10 Mb and have low gene density (Meuleman et al., 2013), whereas variable LADs (vLADs) tend to contain developmentally regulated genes, and their position in the nucleus is altered by developmental cues (Zullo et al., 2012; Harr et al., 2015). Interestingly, many vLADs are enriched for GAGA motif containing sequences, called Lamina Associated Sequences (LASs), which are important for positioning regions of the genome at the nuclear periphery, as shown by experiments that artificially integrated LASs randomly throughout the genome (Zullo et al., 2012; Harr et al., 2015). LASs can be recognized by specific proteins that function to shape LAD architecture or recruit proteins such as the Polycomb Repressor Complex 2 (PRC2), which mediates the repressive H3K27me3 mark (Zullo et al., 2012; Harr et al., 2015). As expression of PRC proteins are deregulated in several cancer types (Luperchio et al., 2014), it is an intriguing possibility that LAD mediated targeting of PRC2 to tumor suppressors could cause their repression in cancer.

The borders of LADs are enriched in binding sites for the chromatin organizer CCCTC- binding factor (CTCF) which, along with SMC- family complex- Cohesin binds to the CTCF recognition motif across the genome and acts as an insulator, thus regulating genomic stability, tissue- specific expression and overall epigenetic homeostasis (Ong and Corces, 2014). CTCF binding sites are found in the borders of both LADs and Topologically Associating Domains (TADs) (Luperchio et al., 2014). While LADs comprise heterochromatic regions, TADs can either be A-type (open, gene-rich chromatin) or B- type (closed, gene - poor) (Achinger-Kawecka and Clark, 2017). While TADs are fairly stable between different cell types, changes within TADs occur during development and differentiation (Luperchio et al., 2014). The relationship between LADs and TADs and the relevance to cancer is an area of active investigation.

### LOCKs

Large Organized Chromatin Lysine Modifications were first described as large heterochromatic domains enriched for H3K9me2 and associated with repressive heterochromatin (Wen et al., 2009). LOCKs comprise > 45% of the genome of liver cells, and 30% of differentiated ES cells, whereas less than 5% of genome is defined as a LOCK in undifferentiated ES cells (Wen et al., 2009). More recent studies also describe blocks of other repressive histones (H3K9me3 and H3K27me3), which occur at negligible levels in ES cells but expand during differentiation, supporting the general model of LOCKs/ blocks as highly dynamic heterochromatic domains during differentiation (Hawkins et al., 2010; Peric-Hupkes et al., 2010). Studies investigating the relationship between LOCKs and gene expression have shown that in liver cells, genes localized within LOCKs are generally silenced, while the same genes were found outside LOCKs in the brain and were expressed. Also, many genes that were not expressed were encompassed by LOCKs in both tissues, showing that LOCKs are strongly correlated with tissue-specific gene silencing (Wen et al., 2009; McDonald et al., 2011).

Interestingly, there is a high degree of overlap between LADs and LOCKs (Wen et al., 2009; McDonald et al., 2011). However, the functional relationship between these chromatin domains is unknown. Elegant studies show LAD sequences become localized to the nuclear rim even when integrated into a non-LAD locus, and this positioning within the nucleus is H3K9me2/3 and H3K27me3 dependent (Zullo et al., 2012; Harr et al., 2015). Another study found that when the methyltransferase that deposits H3K9me2/3 (G9a) was suppressed in cancer cells, LADs “loosen,” since they exhibit less heterochromatin at the nuclear rim (Zullo et al., 2012; Harr et al., 2015) suggesting that changing one of these domains may affect the other.

## LOCKs, LADs and DNA Methylation Transform the Cancer Nucleus

Studies on defining the structural and functional relationships between epigenetic marks and nuclear organization are relatively recent, and thus there are few reports of how these work together in cancer. The frequent overlap of H3K9me3 and 5mC (Rose and Klose, 2014) suggests that LADs have heterochromatin promoting epigenetic marks, such that both the nuclear position and the epigenetic decorations dictate expression, however, further studies are required to determine how universal this is. Some exciting new findings linking DNA methylation, histone methylation and the nuclear lamina suggest that LADs, LOCKs and PMDs overlap in cancer cells reflecting broad reorganization of the genome in the nucleus (Timp and Feinberg, 2013; Rose and Klose, 2014).

In many types of cancer, widespread rearrangement or loss of LOCKs and LADs has been reported (Wen et al., 2009; Berman et al., 2011; Hansen et al., 2011; McDonald et al., 2011). Additionally, the large blocks of hypomethylated DNA found in cancer cells correspond to LADs and LOCKs (Berman et al., 2011; Hansen et al., 2011, 2014; Timp and Feinberg, 2013; Timp et al., 2014). A study comparing colorectal adenocarcinomas to normal tissues from the same patients supports this model: 30% of the genome had profound DNA hypomethylation in large blocks corresponding to LADs and LOCKs (Hansen et al., 2011). This changed the sharply delimited methylation boundaries at CpG islands and shores, creating novel hypomethylation domains in CG-dense regions (Berman et al., 2011). In particular, the PMDs that characterize the repatterning of the cancer methylome are integrated with the changes in nuclear organization (Feinberg et al., 2016). One study found euchromatin islands within LAD/LOCK regions enriched for DNase hypersensitive sites and differentially methylated (Wen et al., 2012) suggesting that the very nature of LOCKs and LADs as primarily repressive domains is redefined in cancer. Since these domains are defined by the method used to isolate them: LADs by association with the nuclear lamina (Guelen et al., 2008), and LOCKs by a long stretch of H3K9Me2 (Wen et al., 2009), and these marks often overlap (Luperchio et al., 2014), it is possible that these are not entirely two different domains of chromatin, but instead reflect approaches that identify the same genomic architectural feature. This suggests that the differences in LADs and LOCKs between cancer and normal cells reflect an extensive reorganization of nuclear and genome structure.

How do changes to chromatin structure impact the cancer cell phenotype? One possibility is a direct impact on gene expression. Recently, it was discovered that PMDs in cancer contain nearly one third of the Transcription Start Sites (TSS) and correspond to LADs and LOCKs (Berman et al., 2011). Interestingly, nearly all the regions of CGIs hypermethylation are encased within large regions of hypomethylation, so that average levels of methylation are the same across the region (**Figure 1**). This essentially eliminates the rationale to focus on small regions of hypermethylation as candidate gene regulatory domains. Indeed, one exciting study has shown that genes with promoters which have the most random (or disordered) methylation pattern are generally not expressed or show wide divergence in expression across samples (Landau et al., 2014). Instead, these large PMDs or regions of disordered methylation that overlap with LOCKs and LADs likely change the 3D organization of chromatin, moving genes within them to the nuclear periphery and silencing them These wide scale changes can result in entire regions of the genome moving from repressive domains to accessible domains, potentially changing their transcriptional status by the fact of their position, but not directly because the methylation at specific CpGs in the genes are altered.

The finding that methylation changes appear even before cancer development and that the methylome is progressively changed as cancer progresses (Feinberg and Vogelstein, 1983a,b; Hansen et al., 2011) supports this as a potential cause of cancer. Additionally, largely euchromatinized DNA is more prone to breaks (Falk et al., 2008). Moreover, it is proposed that the reduction to 50% methylation in PMDs suggests that methylation becomes disordered, with a stochastic methylation of every CpG in every cancer cell which is indicative of cancer heterogeneity.

## Summary and Future Direction

While the changes in nuclear structure in cancer have been appreciated for decades, studies integrating nuclear organization and cancer epigenetics are relatively new and it remains unknown how the epigenetic landscape influences the three dimensional organization of the cancer nucleus. Although DNA hypomethylation and massive changes to histone marks and gene positioning characterizes most cancer cells, how these changes occur is not known. New perspectives on the genome organization in cancer requires adjustment of the model where epigenetic changes at discrete loci are interrogated to find their direct role as a regulator of cancer gene expression. Changes in chromatin structure may contribute to the genomic instability that is a hallmark of most cancers, yet the mechanism of this important change is not yet clear. Finally, how other aspects of the epigenome, including the distribution of histone variants and other epigenetic features, interact with the LADs, LOCKs and PMDs remains an important focus for future study.

## Author Contributions

BM and KS together researched and authored this mini review titled “DNA Methylation, Nuclear Organization and Cancer” for the Epigenomics and Epigenetics special topic “Design and principles of nuclear structure.”

## Conflict of Interest Statement

The authors declare that the research was conducted in the absence of any commercial or financial relationships that could be construed as a potential conflict of interest.
